# SLIM: Simultaneous Logic-in-Memory Computing Exploiting Bilayer Analog OxRAM Devices

**DOI:** 10.1038/s41598-020-59121-0

**Published:** 2020-02-13

**Authors:** Sandeep Kaur Kingra, Vivek Parmar, Che-Chia Chang, Boris Hudec, Tuo-Hung Hou, Manan Suri

**Affiliations:** 1 Department of Electrical Engineering, Indian Institute of Technology-Delhi, Hauz Khas, New Delhi, 110016 India; 2 Department of Electronics Engineering and Institute of Electronics, National Chiao Tung University, Hsinchu, 300 Taiwan

**Keywords:** Electrical and electronic engineering, Nanoscale materials

## Abstract

von Neumann architecture based computers isolate computation and storage (i.e. data is shuttled between computation blocks (processor) and memory blocks). The to-and-fro movement of data leads to a fundamental limitation of modern computers, known as the *Memory wall*. Logic in-Memory (LIM)/In-Memory Computing (IMC) approaches aim to address this bottleneck by directly computing inside memory units thereby eliminating energy-intensive and time-consuming data movement. Several recent works in literature, propose realization of logic function(s) directly using arrays of emerging resistive memory devices (example- memristors, RRAM/ReRAM, PCM, CBRAM, OxRAM, STT-MRAM etc.), rather than using conventional transistors for computing. The logic/embedded-side of digital systems (like processors, micro-controllers) can greatly benefit from such LIM realizations. However, the pure storage-side of digital systems (example SSDs, enterprise storage etc.) will not benefit much from such LIM approaches as when memory arrays are used for logic they lose their core functionality of storage. Thus, there is the need for an approach complementary to existing LIM techniques, that’s more beneficial for the storage-side of digital systems; one that gives compute capability to memory arrays not at the cost of their existing stored states. Fundamentally, this would require memory nanodevice arrays that are capable of storing and computing simultaneously. In this paper, we propose a novel ‘Simultaneous Logic in-Memory’ (SLIM) methodology which is complementary to existing LIM approaches in literature. Through extensive experiments we demonstrate novel SLIM bitcells (1T-1R/2T-1R) comprising non-filamentary bilayer analog OxRAM devices with NMOS transistors. Proposed bitcells are capable of implementing both Memory and Logic operations simultaneously. Detailed programming scheme, array level implementation, and controller architecture are also proposed. Furthermore, to study the impact of proposed SLIM approach for real-world implementations, we performed analysis for two applications: (i) Sobel Edge Detection, and (ii) Binary Neural Network- Multi layer Perceptron (BNN-MLP). By performing all computations in SLIM bitcell array, huge Energy Delay Product (EDP) savings of ≈75× for 1T-1R (≈40× for 2T-1R) SLIM bitcell were observed for edge-detection application while EDP savings of ≈3.5× for 1T-1R (≈1.6× for 2T-1R) SLIM bitcell were observed for BNN-MLP application respectively, in comparison to conventional computing. EDP savings owing to reduction in data transfer between CPU ↔ memory is observed to be ≈780× (for both SLIM bitcells).

## Introduction

**Figure 1:**
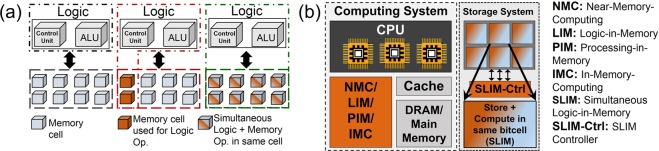
(**a**) von Neumann architecture with separate processing and memory units, LIM: memory blocks can perform logic, and SLIM: all memory blocks capable to perform Storage and Logic operations simultaneously and non-destructively, (**b**) Ideal computing system with LIM/SLIM co-existing with von Neumann CPU, enabling computation at all levels of memory hierarchy.

Over the past few decades, the performance gap between the computing units (where the data is processed) and memory units (where the data is stored) has increased, popularly known as *Memory wall*^1^. It is observed that for many computing tasks, most of the time and energy is consumed in data transfer between the processing unit and memory unit, rather than the computation itself^2^. As a result, bytes per FLOP (memory bandwidth per processor floating point operation) has been decreasing over last five decades^3^. The coalescence of increased data volumes and the need to execute data-intensive applications with limited compute capability and locality are making this imbalance even more challenging today. To tackle these challenges, various solutions have been proposed, from device to system architecture level. Cache hierarchies have been proposed that helped in improving memory access latency at the cost of introducing complexities such as cache coherence and security flaws^3^. Other measures include the extensive use of spatial architectures (distributed on-chip memory that is closer to the computation unit) enabling parallelism using vector processing unit, with large number of cores^4^. Furthermore, accelerators have been designed to match the exact data flow for specific computing algorithms^5^. Three-dimensional memories, commercialized as hybrid memory cube^6^ and high bandwidth memory^7^ chips have been proposed to meet the requirements of high data transfer rate and high memory density. These deliver an order of magnitude higher bandwidth and reduce access energy by up to 5× relative to existing 2-dimensional DRAMs^8^. Moving further, emerging non-volatile memories (NVM) have been introduced into the traditional memory hierarchy to minimize the ‘gap’ between computing and the data units^9^. Promising solutions to eliminate such bottlenecks are governed by ‘Logic in-Memory’ (LIM)/‘Processing in-Memory’ (PIM) approaches^10–22^, where computations are carried out *in*-*situ*, exactly where the data is located^23^. In these approaches^10–22^, the main emphasis was to store input variables or/and logic output in a memory bitcell (see Table 1). LIM offers a clear advantage by reducing latency and energy burdens of the *memory wall*. These approaches still use the conventional von Neumann architecture however it tries to place computation in physical vicinity to the data (generally in main memory). This co-location of memory/data and computation (using logic arrays) makes the process faster. In-storage computing has also been proposed^24^, but in that case also storage memory (HDD/SSD) is treated as just only-storage and a processor is placed next to it. As a result this unit itself acts like a mini computer. Using the high density storage memory itself for computation will make a fundamental or ground-breaking impact in mitigating von Neumann bottleneck. Furthermore, placing computing resources only in cache/main memory does not address the emerging big data challenges, where datasets are too large to fit in main memory. To address this, we propose a novel ‘Simultaneous Logic-in-Memory’ (SLIM) methodology which can be used effectively at all levels of memory hierarchy. In existing LIM approaches, whenever a particular Memory cell is performing a Logic operation it cannot be used for its inherent memory or storage functionality simultaneously, whereas in SLIM the same Memory cell can be used for both Storage function and Logic function simultaneously in space (silicon area) and time (clock cycles). In the proposed SLIM methodology (Table 1), input variables are not stored in the bitcell, rather the initial Memory state is preserved. Figure 1(a) shows concept of different architectures: von Neumann architecture that is used in present day computers, recent LIM architecture and the proposed SLIM architecture. Figure 1(b) shows separation between processor and memory/storage (von Neumann architecture). It also illustrates the concept of an ideal computing system with both LIM and SLIM that enables compute capability at every level of memory hierarchy.

**Table 1:** Salient Features of different LIM techniques proposed in literature.

Ref	Building Block	Logic	Salient Features
^10^	BRS^a^ and CRS^b^ cells	Non-Stateful^c^ V-R logic (sequential logic)	14 out of 16 Boolean function realized in maximum 3 cycles, retains logic output, destructive read operation
^11^	1 BRS	Non-Stateful V-R logic (sequential logic)	all 16 Boolean function realized in maximum 3 cycles, retains logic output, needs rectifying behavior
^12^	1 CRS	Non-Stateful V-R logic (sequential logic)	all 16 Boolean function realized in maximum 3 cycles, retains logic output, needs rectifying behavior
^13^	2 BRS (connected anti-serially)	Non-Stateful V-R logic (sequential logic)	all 16 Boolean function realized in max. 3 cycles, retains logic output
^14^	3 BRS	Stateful^d^ R-R Logic	5 basic Boolean functions can be realized in 2 cycles, retains input variables and logic output
^15^	1T-1R	Non-Stateful V-R logic (sequential logic)	all 16 Boolean function realized in 2 cycles (+1 read cycle), retains input variables and logic output
^16^	3 BRS + passive resistor	Stateful R-R Logic	NAND, AND and other logics can be realized using different number of BRS devices or cascading, retains both input variables and logic output
^17^	3 BRS + passive resistor	Stateful R-R Logic	Boolean function except XOR and XNOR in 1 cycle, retains input variables and output
^18^	1R (4 resistance states) + passive resistor	Stateful R-R Logic	all 16 Boolean operations are realized, retains logic output
This Work	2T-1R or 1T-1R (R:4 resistance states)	Non-Stateful V-R logic	5 basic Boolean functions in 1 cycle, retains initial Memory state and logic output (not storing input variables)

^a^Bipolar restive switches, ^b^Complementary resistive switches, ^c^input (voltage signal) and output (resistance) are in different forms, ^d^input and output are in the same form i.e. either both are voltage signals or recorded in terms of device resistance.

However, this new SLIM architecture requires computational memory devices that can both store data and compute at the same time, usually by device physics or other physical laws. Due to their unique properties, emerging NVMs (such as phase change memories (PCM), oxide based RAM (OxRAM), spin transfer torque MRAM (STT-MRAM), etc) have proved themselves as promising technologies for LIM/SLIM approaches. They offer non-volatility along with a large resistance window supporting clear discrimination of states ‘1’ and ‘0’ (few offer multilevel capability (MLC) as well). Among these candidates, OxRAM is a highly appealing contender because of its scalability, CMOS compatibility, low power dissipation, high operation speed and large endurance. Bilayer OxRAM devices are known to exhibit excellent MLC behaviour owing to their non-filamentary switching nature^25^. Exploiting these attributes of analog bilayer OxRAM devices we propose 1T-1R/2T-1R SLIM bitcells and a novel programming methodology. We show that using our SLIM approach with analog OxRAM it is possible to simultaneously- (i) perform Logic operation *in*-*situ*, (ii) store output of the Logic operation, (iii) preserve the previously stored Memory state (i.e. value stored on the cell prior to the Logic operation) and (iv) read both final logic output and stored Memory values, all while using the same bitcell.

## Experimental Results

### OxRAM Device Fabrication and Characterization

#### SLIM Bitcell

**Figure 2:**
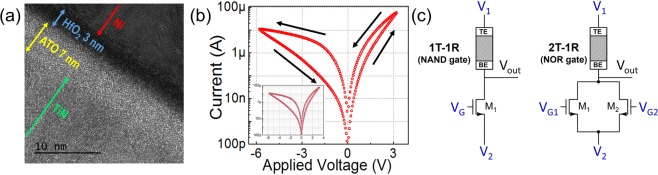
(**a**,**b**) HR-TEM and DC IV curve of bilayer Ni/HfO_2_/ATO/TiN OxRAM device, fabricated for this study (inset shows quite stable repeatable switching for 30 cycles) (**c**) Proposed circuit schematic of 1T-1R/2T-1R SLIM bitcell.

DC IV curve for standalone OxRAM device fabricated (Figure 2(a)) for SLIM bitcell is shown in Figure 2(b). Owing to its non-filamentary, analog resistive switching, the stack exhibits highly reproducible behavior where the resistance can be gradually tuned in a continuous manner by an applied voltage stimuli, as shown in Supplementary Figure S1. The I_*D*_-V_*DS*_ and I_*D*_-V_*GS*_ characteristics of the NMOS transistor are shown in Supplementary Figure S2. Figure 2(c) shows our proposed 1T-1R and 2T-1R SLIM bitcells. BE (V_*out*_) of OxRAM is connected to NMOS drain terminal(s), and TE (V_1_) is used for applying direct programming signals (P1/P2). The gate terminal(s) of NMOS transistor(s) (V_*G*_ for 1T-1R; V_*G*1_ and V_*G*2_ for 2T-1R) are used to load operands as control signals during Logic operations. Body terminal(s) of NMOS transistor(s) is(are) grounded (not shorted to the source terminal). NMOS transistor(s) help with: (i) SLIM realization, (ii) current-compliance (for filamentary OxRAM devices), and (iii) cell selection. In SLIM bitcell, for OxRAM device SET, we use V_*G*_ = 4 V, corresponding to R_*NMOS*_ ≈ 1 kΩ and for RESET we use V_*G*_ = 10 V. High V_*G*_ is required in our bitcell as the OxRAM device dimensions are large and off-chip discrete NMOS transistors are used.

#### Concept of SLIM

**Figure 3:**
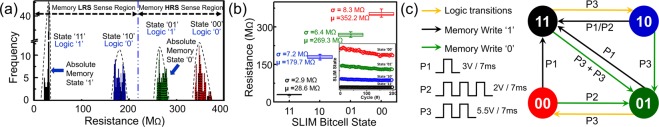
(**a**) SLIM state assignments for Logic and Memory operation. Note Memory LRS and HRS sense regions. Histograms show resistance distributions for 4 selected SLIM states on 1T-1R/2T-1R SLIM bitcell (>100 trials). (**b**) Resistance distribution for 4 selected SLIM states. Inset shows endurance for states: ‘11’, ‘10’, ‘01’, ‘00’ for 200 cycles. (**c**) Proposed SLIM programming signals and Memory-Logic state transitions for 1T-1R/2T-1R SLIM bitcell. All further SLIM operations use P1/P2/P3 pulses at V_1_/V_2_.

SLIM fundamentally differs from other LIM architectures previously reported in literature^10–15^; as it allows the use of a given Memory cell, to simultaneously perform a Logic operation while retaining its actual Memory state. To achieve simultaneous non-destructive Logic operation we propose the following methodology- First, we pick 4 distinct resistance states (labelled: ‘11’, ‘10’, ‘01’ and ‘00’ in Figure 3(a)) from the continuum of attainable OxRAM resistance levels (Supplementary Figure S1). The distinct states are selected on basis of: (i) programming reproducibility, (ii) ease of inter-state transitions between the 4 levels; i.e. requirement of fewer types of programming pulses (signals P1, P2, P3 in Figure 3(c)), and (iii) compatibility for a simultaneous Memory/Logic- Read operation. Each of the 4 selected states (‘11’, ‘10’, ‘01’, ‘00’) are assigned both- Logic (‘1’/‘0’) and Memory (LRS/HRS) definitions respectively (see Figure 3(a)). The sense amplifier threshold and Memory state sensing window is defined such that 2 states lie in Memory LRS sense region while other 2 in Memory HRS sense region respectively. In Figure 3(a), states ‘11’ and ‘10’ lie in Memory LRS region and are assigned Logic ‘1’ and Logic ‘0’ values respectively. While states ‘01’ and ‘00’ lie in Memory HRS region and are assigned Logic ‘1’ and Logic ‘0’ values respectively. Thus, with such state assignment, the system has two representations each for; Logic ‘1’, Logic ‘0’, Memory HRS and Memory LRS. While executing any Logic operation, the SLIM programming scheme permits state transitions only within the Logic (‘1’/‘0’) levels of a particular Memory sense region (i.e. Logic ‘1’ ↔ ‘0’ within HRS or within LRS sense regions are permitted, but Logic ‘1’ ↔ ‘0’ through HRS ↔ LRS is not permissible). Thus any initially stored Memory state on the bitcell can be preserved even after executing a Logic operation. A single Read operation can simultaneously read and decode both Memory- and Logic values on the device.

#### SLIM State Definitions

To account for device variability, we have considered a reliable resistance range and resistance window for 4 SLIM states. Following are the resistance ranges of each SLIM state:

- **State** ‘**11**’**:** The resistance of device ranges from 20 MΩ to 33 MΩ with mean (*μ*) = 28.69 MΩ. This is *absolute Memory state* ‘*1*’ i.e. during Memory Write ‘1’ operation device is programmed to this resistance state.
- **State** ‘**10**’**:** The resistance of device ranges from 170 MΩ to 190 MΩ with *μ* = 179.74 MΩ.
- **State** ‘**01**’**:** The resistance of device ranges from 260 MΩ to 280 MΩ with *μ* = 269.36 MΩ. This is *absolute Memory state* ‘*0*’ i.e. during Memory Write ‘0’ operation device is programmed to this resistance state.
- **State** ‘**00**’**:** The resistance of device ranges from 340 MΩ to 360 MΩ with *μ* = 352.17 MΩ.

Figure 3(b) shows experimentally programmed distributions of the 4 SLIM resistance levels and their cycling endurance. The endurance test was conducted using constant-voltage stress (CVS) program. Four SLIM states were achieved using programming signals (P1/P2/P3) in one sequence, and the sequence was repeated over 200 times to test the endurance. The state diagram for SLIM methodology is shown in Figure 3(c). It illustrates the required programming signals (P1, P2, P3) and possible SLIM inter-state transitions while implementing any Logic or Memory operation. On application of P1/P2 pulses, the OxRAM device’s resistance is gradually lowered while with application of P3 pulse the resistance is increased.

### Experimental validation

#### Memory-Write Operation

**Figure 4:**
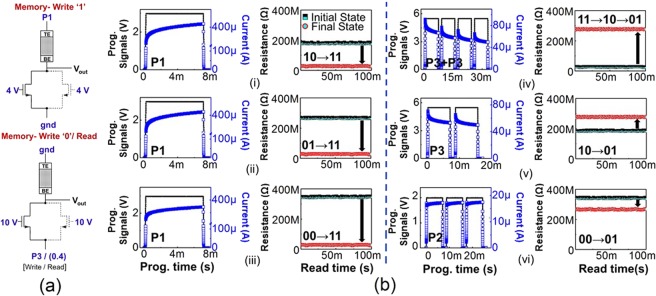
(**a**) Applied example signals for Memory Write ‘1’ and Memory Write ‘0’. Read conditions are specified in brackets.[All pulse duration = 7 ms] (**b**) Experimental measurements for Memory Write ‘1’ operation (program device to state ‘11’) with device’s initial state as: (i) ‘10’, (ii) ‘01’, and (iii) ‘00’. Memory Write ‘0’ operation (program device to state ‘01’) with device’s initial state as (iv) ‘11’, (v) ‘10’ and (vi) ‘00’. Blue line: transient current through OxRAM device. Black line: P1/P2/P3 (applied signals). Black square: Initial resistance state, Red circle: Final resistance state post SLIM operation. Please note in (iv,v), the transient current through OxRAM device falls due to gradual increase in non-volatile resistance with application of successive reset pulses. **Note:** *The current scale in (i*–*vi) is varied for clear demonstration of gradual switching in the OxRAM device*.

These refer to purely Storage operations, with the purpose of just storing a bit (‘1’/‘0’) on the SLIM bitcell (1T-1R/2T-1R). Data can be stored on the bitcell through Memory Write- ‘1’ (i.e. absolute Memory state ‘11’, LRS) or Memory Write- ‘0’ (i.e. absolute Memory state ‘01’, HRS). During Memory Write operation, the bitcell is programmed to absolute Memory states (as shown in Figure 3(a,c)). Figure 4(a) shows precise bitcell programming methodology for the Memory Write operations. During Memory Write ‘1’, programming signal P1 is applied at terminal V_1_, terminal V_2_ is grounded and V_*G*_ is kept at 4 V. Whereas in Memory Write ‘0’, terminal V_1_ is grounded, programming signal P3 is applied at terminal V_2_ and V_*G*_ is made 10 V. Figure 4(b) shows detailed experimental results for Memory Write ‘1’ (Figure 4(b)(i–iii)) and Memory Write ‘0’ (Figure 4(b)(iv–vi)) from all possible initial states. We can observe SET switching in Figure 4(b)(i–iii,vi) as the current through the OxRAM device gradually increases during applied programming pulse P1/P2. Similarly, RESET switching is evident in Figure 4(b)(iv,v) as the current through the OxRAM device gradually decreases during applied programming pulse P3. During SET programming ≈100 *μ*A to 450 *μ*A current flows whereas during RESET programming ≈40 *μ*A to 80 *μ*A current flows through the bitcell.

#### 1T-1R SLIM protocol (Universal NAND gate)

**Figure 5:**
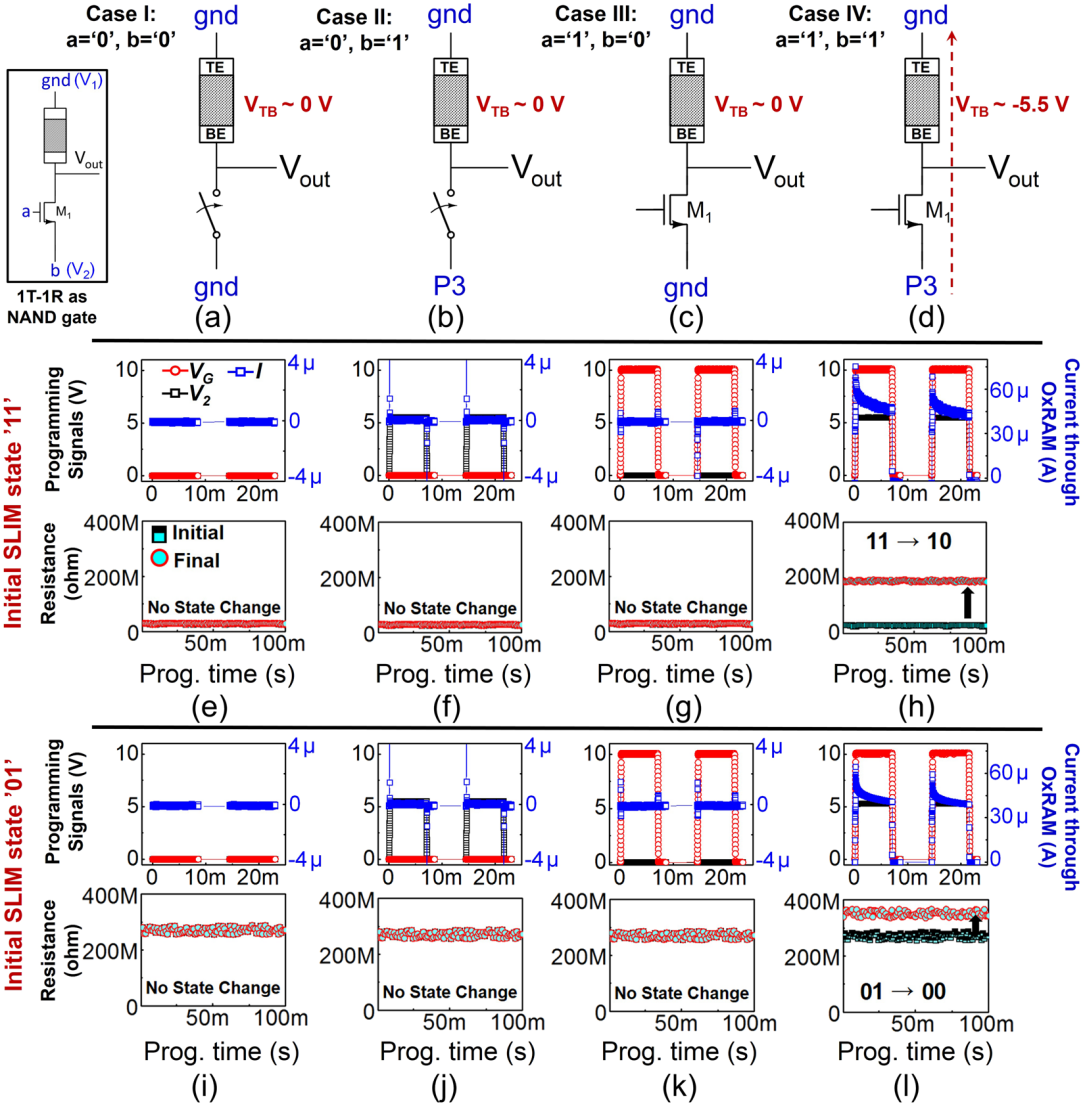
Four possible input operand combinations: (**a**) *a* = *b* = ‘0’; (**b**) *a* = ‘0’, *b* = ‘1’; (**c**) *a* = ‘1’, *b* = ‘0’; (**d**) *a* = *b* = ‘1’; corresponding to NAND truth table and proposed signal mapping for each case for the 1T-1R SLIM bitcell. [V_*TB*_ = V_*TE*_ − V_*BE*_; operand *a* is mapped to V_*G*_ = 10 V (7 ms long); operand *b* is mapped to V_2_ = P3 = 5.5 V (7 ms long)]. Experimental results for NAND logic implemented using 1T-1R SLIM bitcell with device’s initial state: ‘11’ (**e**–**h**), and ‘01’ (**i**–**l**). Among the four operand combinations, OxRAM device switches to Logic HRS state (‘10’ or ‘00’) only for *a* = *b* = ‘1’. Blue: transient current through OxRAM device.

These refer to pure Logic operations to be performed on the 1T-1R SLIM bitcell (ex- NAND, OR, NOT etc.). Table 2 presents the SLIM state-mapping and truth-table for realizing a 2-input NAND gate operation using a single 1T-1R SLIM bitcell. Prior to executing Logic operation the bitcell may initially contain any stored Memory state (i.e. absolute Memory state ‘11’ or ‘01’), as a consequence of a preceding Memory operation(s). While realizing Logic operation, SLIM bitcell may undergo a state change but the overall Memory state is preserved. Table 2 shows that previous Memory state is effectively preserved after each NAND Logic operation, by the virtue of SLIM state assignment and programming methodology described in Figure 3(a,c). The circuit schematic and NAND programming signals for all possible 1-bit, 2-input (*a*, *b*) operand combinations are shown in Figure 5(a–d). Operands *a*/*b* are mapped to V_*G*_/V_2_ terminal of bitcell respectively, keeping V_1_ grounded. The voltage conditions for operand mapping are as below:

- ***a*** = ***b*** = ‘**0**’**:** In this case, both V_*G*_ = V_2_ = 0 V (gnd).
- ***a*** = ‘**0**’**;** ***b*** = ‘**1**’**:** In this case, V_*G*_ = 0 V (gnd) whereas V_2_ = 5.5 V.
- ***a*** = ‘**1**’**;** ***b*** = ‘**0**’**:** In this case, V_*G*_ = 10 V whereas V_2_ = 0 V (gnd).
- ***a*** = ‘**1**’**;** ***b*** = ‘**1**’**:** In this case, V_*G*_ = 10 V whereas V_2_ = 5.5 V. In this case, the device will undergo RESET programming.

**Table 2:** Two-input NAND logic Gate truth-table using our 1T-1R SLIM methodology.

Variables		Stored Memory state: 1			Stored Memory state: 0		
*a*	*b*	Device state		Logic output	Device state		Logic output
		Initial	Final		Initial	Final	
0	0	11	11	1	01	01	1
0	1	11	11	1	01	01	1
1	0	11	11	1	01	01	1
1	1	11	10	0	01	00	0

When *a* is ‘0’, NMOS transistor is OFF. However, only when both *a* and *b* are ‘1’ (Logic high), the applied programming signal drops across OxRAM and it undergoes RESET switching. Figure 5(e–h) show experimental implementation of NAND Logic operation on SLIM bitcell for initial device state: ‘11’ (i.e. stored Memory LRS/‘1’), and Figure 5(i–l) for initial device state: ‘01’ (i.e. stored Memory HRS/‘0’). It is evident that for two consecutive identical RESET pulses (P3 at terminal V_2_, operand *b* = ‘1’), owing to non-filamentary switching, the OxRAM resistance gradually increases and the current flowing through the device decreases. Similar OxRAM behaviour in response to consecutive RESET pulses (P3) was also observed while performing SLIM Memory operations (Figure 4(b)). All experiments repeatedly validate preservation of initial Memory state post Logic operation.

#### 2T-1R SLIM protocol (Universal NOR gate)

**Figure 6:**
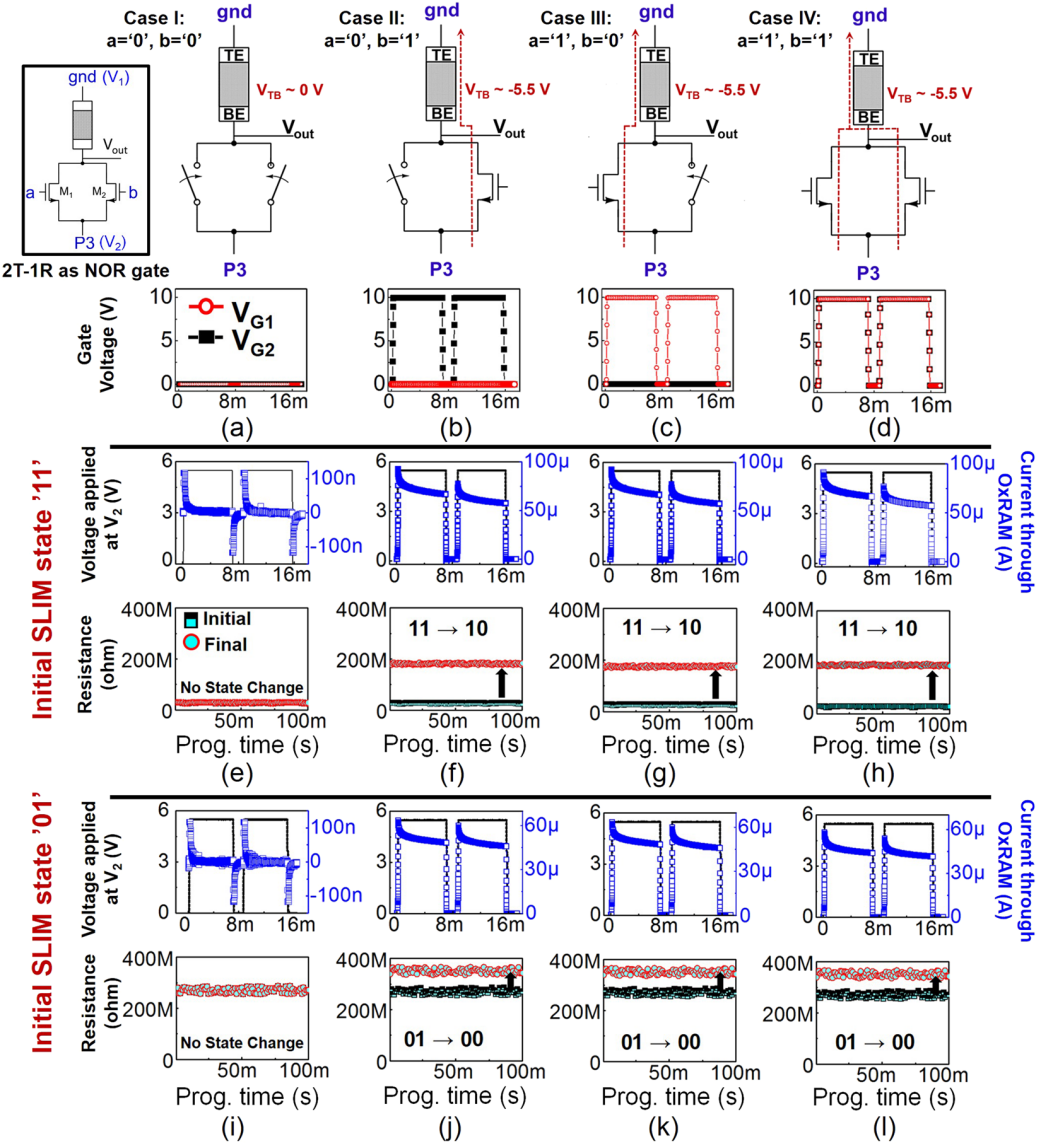
Four possible input operand combinations: (**a**) *a* = *b* = ‘0’; (**b**) *a* = ‘0’, *b* = ‘1’; (**c**) *a* = ‘1’, *b* = ‘0’; (**d**) *a* = *b* = ‘1’; corresponding to NOR truth table and proposed signal mapping for each case for the 2T-1R SLIM bitcell. [V_*TB*_ = V_*TE*_ − V_*BE*_; V_*G*_ = 10 V (7 ms long); V_2_ = P3 = 5.5 V (7 ms long). Experimental results for NOR logic implemented using 2T-1R SLIM bitcell with device’s initial state: ‘11’ (**e**–**h**), and ‘01’ (**i**–**l**). Among the four operand combinations, OxRAM device switches to Logic HRS state (‘10’ or ‘00’) for *a* = ‘0’, *b* = ‘1’; *a* = ‘1’, *b* = ‘0’ and *a* = *b* = ‘1’. Blue: transient current through OxRAM device. Black line: P3 in all cases (applied signal).

A single 2T-1R SLIM bitcell is capable of performing multiple Logic operations ex- NOR, AND, NOT, NAND, OR etc. An additional transistor compared to 1T-1R bitcell configuration adds more functionality to this bitcell. Table 3 presents the SLIM state-mapping and truth-table for realizing a 2-input NOR gate operation using a single 2T-1R SLIM bitcell. Similar to 1T-1R SLIM bitcell, we assume 2T-1R bitcell to be in absolute Memory state (‘11’ or ‘01’) before executing a Logic operation. Table 3 shows that previous Memory state is effectively preserved after each NOR Logic operation. Operands *a*/*b* are mapped to V_*G*1_/V_*G*2_ respectively, while signal P3 is applied on terminal V_2_, keeping V_1_ grounded. When either *a* or *b* is ‘0’, the corresponding NMOS is OFF (see Figure 6). However, when either *a* or *b* is ‘1’ (Logic high), the corresponding NMOS is ON, as a result the applied programming signal drops across OxRAM and it undergoes RESET switching. Figure 6(e–h) show experimental implementation of NOR Logic operation on 2T-1R SLIM bitcell for initial device state: ‘11’ (i.e. stored Memory LRS/‘1’), and Figure 6(i–l) for initial device state: ‘01’ (i.e. stored Memory HRS/‘0’).

**Algorithm 1:**
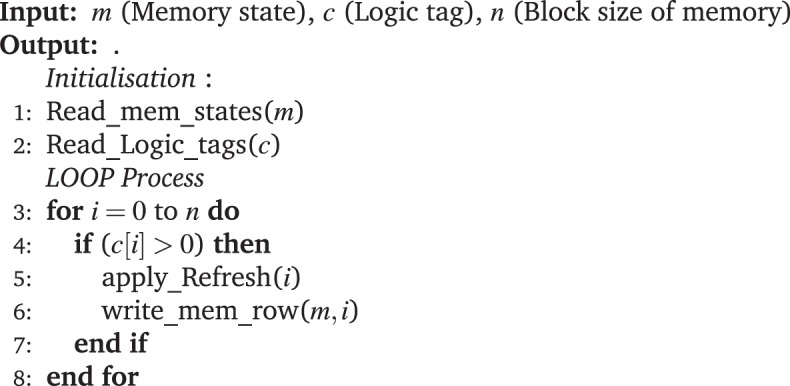
Algorithm for *n* × *n* SLIM bitcell array Refresh.

**Table 3:** Two-input NOR logic Gate truth-table using our 2T-1R SLIM methodology.

Variables		Stored Memory state: 1			Stored Memory state: 0		
*a*	*b*	Device state		Logic output	Device state		Logic output
		Initial	Final		Initial	Final	
0	0	11	11	1	01	01	1
0	1	11	10	0	01	00	0
1	0	11	10	0	01	00	0
1	1	11	10	0	01	00	0

### Array-level Implementation of SLIM

**Figure 7:**
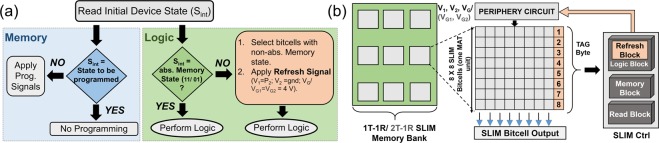
(**a**) Flowcharts for SLIM: Memory Write operation and Logic operations. Intelligent read is performed in both operations. Refresh scheme is an internal part of Logic operation. (**b**) Optimized Refresh Scheme corresponding to multiple SLIM MATs (Matrices). Each SLIM MAT has 8 × 8 bits. 1 Tag register is allocated to each SLIM MAT to track row status. Within each Tag register, 1-bit corresponds to 1 row of 8 × 8 SLIM MAT. Tag Byte is initialized to zero once a fresh SLIM MAT is used. Once a row is used for Logic operation, tag bit corresponding to it, is set high. When the tag byte contain all ‘1’s, the Refresh block is triggered and it sends instruction to refresh the contents of complete SLIM MAT. After Refresh operation, all the SLIM bitcells in given SLIM MAT will have absolute Memory states (‘11’/‘01’).

To avoid possible resistance state saturation during multiple consecutive SLIM Logic ↔ Memory operations inside a large 2T-1R SLIM bitcell array, we define an intelligent SLIM control protocol with an added Refresh block (Figure 7). In Refresh scheme, first the initial state of the bitcell is read. If the bitcell is in absolute Memory state (i.e. ‘11’ or ‘01’), Logic implementation can be performed directly by following programming scheme defined in Figure 3(c). However, in case the state of bitcell is in non-absolute Memory state (i.e. ‘10’ or ‘00’), Refresh is applied (i.e. signal P2 at V_1_, V_2_ = gnd, V_*G*1_ = V_*G*2_ = 4 V). As shown in Figure 3(c), application of P2 always restores the bitcell from a Logic state (‘10’ or ‘00’) to its corresponding absolute Memory state (‘11’ or ‘01’) respectively. This enables the same bitcell for the next Logic operation while effectively preserving the previous Memory state. The Refresh scheme can be further optimized by performing it only periodically at the array level, using Tag-bytes to track the last operation of the SLIM MAT unit (see Figure 7(b) for further details). SLIM MAT indicates 2-dimensional SLIM bitcell array. Once, entire MAT has been used for Logic operations, row-wise Refresh operation may be enabled. The algorithm to explain the Refresh operation at architectural level is summarized in Algorithm 1. As shown in Figure 7(b), a TAG register is allocated for each SLIM MAT with 1-bit corresponding to each row. When bitcells in non-absolute Memory state/row > pre-determined threshold (0.5 × *n* where n = number of bitcells in a row) the corresponding TAG bit is set high. The threshold based triggering of TAG bits ensures that Refresh operations are minimized and in turn helps reduce energy overheads for SLIM bitcell array operation.

**Figure 8:**
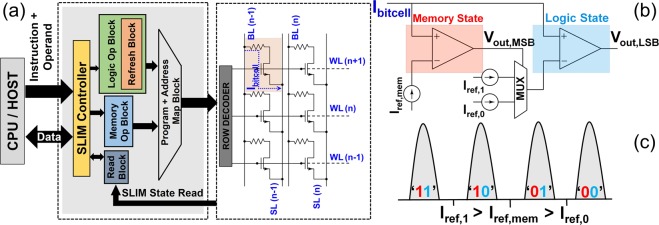
(**a**) Block diagram of SLIM processing unit. Refresh block forms an internal part of the Logic operation block. User operands are passed first to the SLIM control unit. The control unit with other blocks maps Logic operations on the 1T-1R array. (**b**,**c**) Proposed sensing mechanism used for reading the SLIM bitcell state.

Figure 8(a) presents the SLIM controller implementation encompassing all details of Figures 3(a,c) and 7(a). It has three blocks to control Memory Write-, Logic- and Read-operations. There is an additional control signal to choose either Memory/Logic mode. Within the Logic block, an additional intelligence is built to implement refresh mechanism. Figure 8(b,c) illustrates the sensing mechanism used for reading the SLIM bitcell state. The MSB (Most significant bit) and LSB (Least significant bit) of the SLIM state depicts the Memory and Logic state respectively. In case, the application requires only reading the Memory state, V_*out*,*MSB*_ is sufficient, (i.e. comparing I_*bitcell*_ against pre-defined Memory threshold window I_*ref*,*mem*_). To decode the Logic state, an additional comparison with Logic threshold (i.e. I_*ref*,1_, I_*ref*,0_) should be performed. In literature, current sense amplifiers (CSA) for sensing multilevel states have been proposed^26^.

**Figure 9:**
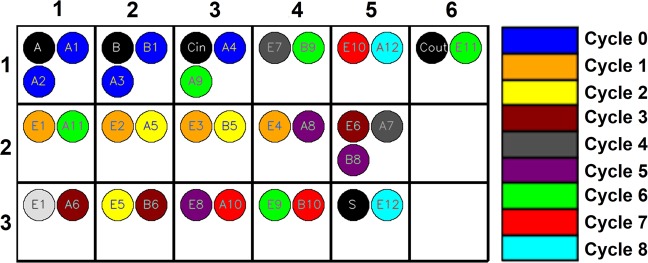
1-bit Full Adder operation mapping on 1T-1R SLIM bitcell array (NAND logic) using SLIM Operation Compiler. Here An, Bn indicate inputs, En indicates output of Logic operation. Black circles indicate input/output for the adder.

Figure 9 shows the implementation of a 1-bit Full Adder (FA) using the proposed 1T-1R SLIM NAND logic with accurate cycle and bitcell location mapping. Mapping has been derived using a customized SLIM bitcell compiler. The main constraints used for deriving the mapping are: (1) Inputs present in a single row, (2) Output not mapped in same row as input, and (3) All operations that use same input and output rows can be performed in parallel. The cycle count indicated in Figure 9 includes: (1) Read cycle for reading from input row, and (2) SLIM Operation cycle to apply appropriate programming signals to the terminals of SLIM bitcell in output row. Table 4 illustrates the extension of SLIM methodology to Logic operations beyond NAND/NOR (while using the same programming signals). It should be noted that in all these cases only one SLIM bitcell is required to realize the Boolean function.

**Table 4:** Program scheme for realizing Boolean operations using a SLIM bitcell.

Logic^a^	*V*_1_	*V*_2_	*V*_*G*1_	*V*_*G*2_
$\overline{{\bf{a}}}$	**gnd**	**P3**	**a**	**a**
$\overline{{\bf{b}}}$	**gnd**	**P3**	**b**	**b**
**a** + **b**	**gnd**	$\overline{{\bf{b}}}$	$\overline{{\bf{a}}}$	$\overline{{\bf{a}}}$
$\overline{a+b}$	gnd	P3	a	b
a.b	gnd	P3	$\overline{a}$	$\overline{b}$
$\overline{{\bf{a}}.\,{\bf{b}}}$	**gnd**	**b**	**a**	**a**

^a^2T-1R bitcell can realize all the Boolean functions listed above whereas 1T-1R can realize only the highlighted functions.

## SLIM Application Analysis

The proposed SLIM bitcell is logically complete as it is possible to realize NAND/NOR gates (i.e. a universal gates). All other basic gates and arithmetic functions can be realized by mapping the desired function on NAND/NOR gates. To study the impact of proposed SLIM methodology on logic computation we derived energy and latency estimates for basic logic gates normalized w.r.t. the values for SLIM based NAND gate (i.e. a single 1T-1R SLIM bitcell) and SLIM based NOR gate (i.e. a single 2T-1R SLIM bitcell) respectively. We used an approximate model for deriving switching energy as shown in Eq. 1 where N denotes total possible input combinations of input *x*_*i*_. Switch_events function is a mathematical model that determines state transition events at each node assuming NAND/NOR based logic for computing the specified Logic operation. Latency is derived by estimating total number of Logic levels required for computing the Logic function based on the computation graph derived using NAND/NOR Logic. Results are summarized in Table 5.

1 $$Energy(SLIM\,op)=\frac{{\sum }_{i}^{N}\,Switch\_events(SLIM\,op,{x}_{i})}{N}$$

**Table 5:** SLIM bitcell count, Normalized Energy/Operation and Normalized latency count for different operations using SLIM NAND/NOR logic gate.

Logic Operation Type	1T-1R (NAND)			2T-1R (NOR)		
	#SLIM bitcells	Norm. Energy/Op	Norm. Latency	#SLIM bitcells	Norm. Energy/Op	Norm. Latency
OR	3	2×	2×	2	1×	2×
AND	2	1×	2×	3	2×	2×
NOR	4	3×	3×	1	1×	1×
NAND	1	1×	1×	4	3×	3×
XOR	4	2×	3×	5	4×	3×
XNOR	5	3×	3×	4	3×	3×
-bit Half Adder	5	2×	3×	5	4×	4×
-bit Full Adder	9	3×	6×	9	6×	6×

(All values normalized w.r.t. SLIM 1T-1R NAND and provide worst-case estimates i.e. maximum device switching).

**Figure 10:**
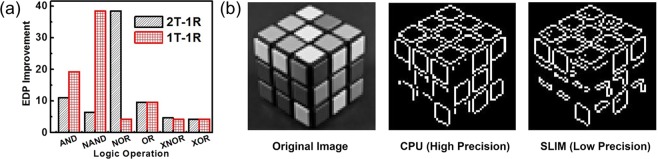
(a) EDP comparison for performing 64-bit Logic operations using SLIM array w.r.t. conventional CPU architecture (fetching operands from DRAM (DDR3)), (b) Edge-detection output of ‘CPU + DRAM’ (center) and ‘CPU + SLIM bitcell array’ (right) along with the original image (left). Image sources: (b) Original image shown on left is a resized and cropped version of “BW Rubik’s Cube” by Gerwin Sturm (https://www.flickr.com/photos/scarygami/5568844961), licensed under CC BY-SA 2.0 (https://creativecommons.org/licenses/by-sa/2.0/). Images in center and right are generated by performing edge detection).

OxRAM devices used in the experimental study have larger area and hence exhibit higher delay and energy costs. In order to perform a fair comparison with state-of-the-art architectures, we have used device parameters of advanced bilayer filamentary HfO_*x*_ device^27,28^ (shown in Supplementary Table S1). Figure 10(a) highlights the performance (EDP) comparison for 64-bit Logic operations performed using a conventional CPU (Intel Core i5-2500 Sandy Bridge CPU running at 3.3 GHz) and SLIM bitcell assuming data is to be fetched from DRAM/SLIM bitcell array. For CPU, latency and energy values from technical reports^29,30^ have been considered. For data transfer, bus-width used for all memory interfaces is 128-bit. For SLIM bitcell based estimates, we incorporated wire delays and dissipation as specified in IRDS guidelines^31^ (Node scaling was used to estimate for 28 nm node starting from 14 nm/16 nm). The energy for 1-bit Logic gates (1-bit operand) has been derived and extrapolated for n-bit logic gates (assuming n-bit operations can be realized as parallel computation using n banks in the SLIM bitcell array). For all given Logic operations, we can observe a minimum EDP benefit of ≈4×. To further analyze the impact of introducing SLIM bitcell array in the memory hierarchy on real-world workloads, we performed two case studies (Edge Detection and BNN).

### Case I: Sobel Edge Detection

An image processing application of ‘edge-detection’ was analyzed. Edge-detection with Sobel operator was used, which involves convolution of 64 × 64 image (shown in Figure 10(b)) with a 3 × 3 sized filter. The 3 × 3 Sobel filter operation involves 9×MUL and 9×ADD operations. To minimize complexity of implementation, 4-bit precision is considered for using SLIM bitcell array in comparison to the full 8-bit precision used by CPU. Carry-Save Architecture (CSA) is used for implementing 4-bit Multiplier. Furthermore, 4-bit ripple carry adder (RCA) is used for calculating the final result. SLIM bitcell array used for the analysis has a size of 4 kB with MAT size of 8 × 8 spread across 16 banks with 32 MATs/bank. Total number of parallel 1-bit SLIM operations possible are 32 × 16 × 8 = 4096. Since all operations are of 4-bit precision, 8 parallel operations can be performed simultaneously in a single MAT. Hence maximum operations that can be performed in parallel across the entire SLIM bitcell array are 128. Based on the mapping, we derived worst case latency and EDP values for the application. For each Sobel operation, the CPU uses 9×IMUL, 9×ADD, 18×LOAD and 9×STORE operations. Cycle latency and average energy dissipation for each operation is used for calculating EDP. Total Sobel operations performed are 64 × 64 = 4096. Figure 10(b) presents the results for edge-detection using high-precision CPU and low-precision SLIM bitcell array respectively. Table 6 summarizes relative EDP comparison in terms of data transfer and computation operations for both approaches. It is assumed that data (for computation) is fetched from the main memory (DRAM array) for the CPU case. Furthermore, energy and latency penalties arising due to cache misses are also taken into account. Instructions for SLIM Logic operations are incorporated within memory instructions to minimize the overhead. By performing all computations inside the SLIM bitcell array, the total EDP for the application reduces up to 75× and 40× for 1T-1R and 2T-1R SLIM bitcell arrays respectively (summarized in Table 6). This performance benefit will be more pronounced with further improvements in device technology and compiler based optimizations. It is also observed from Table 6 that data transfer savings in EDP is ≈780× (both 1T-1R/2T-1R SLIM bitcells) due to reduction in data transfer (CPU ↔ memory) as a result of IMC. For computation using conventional CPU architecture, all operands need to be fetched from DRAM. However for doing computation using SLIM, operands are already present in the SLIM bitcell array (as it resides at the same position as DRAM). Due to this, the CPU needs to send instructions and fetch results.

**Table 6:** Performance results for application benchmarks using conventional and SLIM based system configuration.

Application	System Config	Cell Type	Energy Delay Product (pJs)			Ratio w.r.t. CPU + DRAM
			Data Transfer	Compute	Overall	
Sobel Edge Detection	CPU + DRAM	—	1.31E-01	2.48E+05	2.48E+05	1
	SLIM	1T-1R	1.68E-04	3.31E+03	3.31E+03	75.05
		2T-1R	1.68E-04	6.19E+03	6.19E+03	40.16
BNN-MLP	CPU + DRAM	—	4.82	3.68E+06	3.68E+06	1
	SLIM	1T-1R	6.15E-06	1.09E+06	1.09E+06	3.47
		2T-1R	6.15E-06	2.36E+06	2.36E+06	1.61

### Case II: Binary Neural Network- Multi layer Perceptron (BNN-MLP)

**Figure 11:**
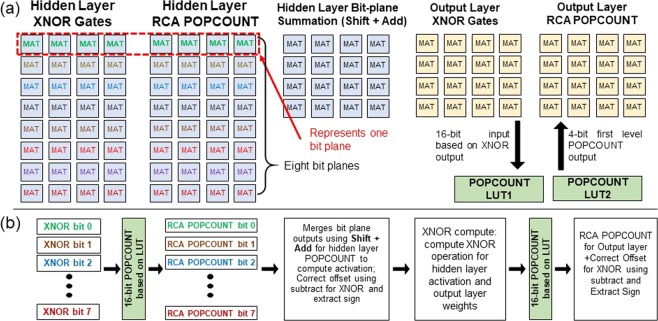
(**a**) Mapping of operations (XNOR and POPCOUNT) over SLIM MATs with addition SRAM based POPCOUNT LUT modules. All Logic operations are mapped on independent bitcells, considering no bitcell is reused during one inference cycle. Mapping ensures proximity for bitcells performing the same type of operation in the same layer. (**b**) Flowchart of SLIM-BNN computation. Since binary weight used for XNOR computation are [0, 1] rather than [−1, 1], a fixed offset has been subtracted in order to compute the same dot product.

EDP analysis was performed for realizing BNN-MLP^32^ network of size 784 × 100 × 10 (shown in Supplementary Figure S4(a)) on SLIM bitcell array (array size = 1 MB realized using 32 × 32 SLIM MATs). The network is trained on MNIST dataset (Supplementary Figure S4(b)). The computation is mapped as XNOR and POPCOUNT operations. Operation mapping is summarized in Figure 11(a) and sequence of operations used for BNN-MLP computation are shown in Figure 11(b). First level of mapping is based on layer association (hidden, output) followed by operation type (XNOR, POPCOUNT). For hidden layer computation, weights are binary (1,0) while the inputs are quantized to 8-bit signed-integers and hence 8 bit-planes were computed simultaneously. For POPCOUNT operation, first stage was implemented using LUT^33^ (power estimates based on literature^34^). Second stage of POPCOUNT is implemented by summation of the intermediate 4-bit results using SLIM MAT based hierarchical adder tree (shown in Figure S4(c)). After POPCOUNT bit-planes are merged by Shift + Add followed by offset correction. Activation of neuron is computed as sign of output. In case of output layer, all computations are performed in a single bit-plane for XNOR followed by similar POPCOUNT operation and offset correction. For our study, the entire computation mapping required 6909 SLIM MATs of size 32 × 32. For mapping BNN-MLP network on CPU following instructions were used: LOAD, STORE, XOR, NOT, POPCNT, SHL (shift logical left), ADD, SUB, CMP. Detailed discussion regarding mapping and operation scheduling is also provided in Supplementary Section S5. Final estimated EDP results are summarized in Table 6. 1T-1R SLIM based implementation of BNN-MLP offered a benefit of ≈3.5× compared to CPU + DRAM whereas 2T-1R based implementation offers a benefit of up to ≈1.6×.

## Discussion

OxRAM devices fabricated for this study are for demonstration purposes, and hence large (50 *μ*m × 50 *μ*m). Further scaled OxRAM devices with integrated on-chip NMOS selectors will lead to significant reduction in operating voltages (SET/RESET). Programming currents in non-filamentary OxRAM devices, like the ones used for this study, have been shown to scale with active area of the device^35^. For filamentary OxRAM devices one would expect variability to become worse with scaling^36^, however in case of non-filamentary OxRAM the homogeneous interfacial switching helps overcome the intrinsic trade-off between operating current and variability^35^. SLIM bitcell is based on V-R logic realization and hence direct cascading of logic gates is not possible. In this case study, OxRAM device exhibits higher resistance levels due to the choice of read voltage levels, which would require precise sense amplifiers to accurately determine the SLIM bitcell state leading to additional overhead in the periphery. The sense-amplifier constraints may be relaxed by tuning the resistance window of the OxRAM devices. Resistance window tuning can be achieved in multiple ways such as material/interface stack engineering, and choice of different programming and read voltage levels. In addition to this, for Logic realizations using SLIM methodology, periodic Refresh operation is required that brings added overhead to SLIM controller. However, for high density storage systems, the frequency of Refresh operation will decrease and optimization in Refresh scheme can further address this issue. SLIM bitcell operation relies on using WRITE operations for performing logic and hence faces a fundamental limitation due to limited endurance of OxRAM devices. We have provided an additional analysis with discussion on strategies to address this issue in the supplementary material (see Supplementary Section S6). The analysis validates that the endurance requirement for a single BNN-MLP inference operation is well within the endurance limit of typical OxRAM devices reported in literature^37^. For the best case presented in our analysis (shown in Supplementary Figure S5), every individual operation is mapped on a distinct SLIM bitcell. Using this strategy, ≈10^12^ inference operations can be performed with the device endurance (proposed in literature^38^) consuming a computational memory of ≈7 Mb. Since the proposed SLIM approach is targeted for high density storage systems (large SSDs with capacities >100 s of GBs/TBs), access to a large number of SLIM bitcells can be assumed for sparse mapping of Logic operations. This would minimize the overall endurance requirement.

## Conclusion

We presented a novel ‘Simultaneous Logic-in-Memory’ methodology (SLIM) to overcome the von Neumann bottleneck. This methodology allows memory bitcell to be used for computation without losing the previously stored Memory state by exploiting the analog behaviour of bilayer OxRAM devices. By using 4 distinct Memory states of OxRAM device, we have experimentally validated NAND/NOR logic on 1T-1R/2T-1R SLIM bitcell and also showed realization of multiple complex Logic functions. We further enhanced the scope of SLIM bitcell by proposing programming scheme for realizing other Boolean operations apart from NAND/NOR gate using a single SLIM bitcell. We also proposed a SLIM controller design and Refresh scheme for realizing consecutive Logic operations. We compared the performance of SLIM methodology with conventional CPU + memory implementations for two applications: (i) Sobel Edge Detection (ii) BNN-MLP. The analysis showed huge EDP savings ≈75× for 1T-1R (≈40× for 2T-1R) SLIM bitcell for an edge-detection application while EDP savings of ≈3.5× for 1T-1R (≈1.6× for 2T-1R) SLIM bitcell for BNN-MLP application. Using this proposed scheme, a high density storage can act as free computational resource without disturbing the user’s stored data.

## Methods

### Device Fabrication

Analog resistive switching OxRAM stacks of Ni/3 nm *HfO*_2_/7 nm Al-doped-*TiO*_2_ (ATO)/TiN (top to bottom) structure were fabricated by following a CMOS compatible process. The active device area was 50 *μ*m × 50 *μ*m, and the ATO as well as HfO_2_ were deposited using plasma-enhanced atomic layer deposition (PE-ALD). Transmission electron microscopy (TEM) cross-section image of the device stack is shown in Figure 2(a), where amorphous dielectric bilayer is seen deposited on the TiN bottom electrode (BE) film with high uniformity, a fingerprint of ALD. The device fabrication flow is as follows: First, 100 nm thick TiN BE film was deposited on thermal-*SiO*_2_ (500 nm)/Si wafer by physical vapor deposition (PVD), RF magnetron sputtering. The BEs were then patterned by optical photolithography (first mask) and dry etching using inductively-coupled plasma (ICP). The bottom, 7 nm thick ATO dielectric, was then deposited by interchanging varying amount of *TiO*_2_ and *Al*_2_*O*_3_ PE-ALD cycles, using TDMATi (Tetrakis(dimethylamido)titanium) and TMA (trimethylaluminum) as metal-organic precursors and O_2_ plasma as a reactant. Upper, 3 nm thick dielectric *HfO*_2_ film, was deposited using TDMAHf (Tetrakis(dimethylamido)hafnium) and *O*_2_ plasma. All depositions were carried out at 250 °C using Veeco-CNT Fiji F202 remote plasma hot-wall reactor PE-ALD system. Top Electrode (TE) pattern (similar to the BE pattern but rotated 90°) was defined using second optical photo-lithography mask and 100 nm thick Ni TE film was deposited by DC sputtering and patterned using lift-off technique. Final photolithography (third mask) and ICP dry etching step was performed to open the contact windows (etch the dielectrics) to the BE contact pads. Wire bonding and packaging were the final steps for the OxRAM encapsulation.

### Electrical measurements for SLIM bitcell characterization

We used Keithley 4200 SCS parameter analyzer with custom setup (Supplementary Figure S3) to perform electrical measurements. Keithley 4210 high power SMU (source measure unit) and Keithley 4225 PMU (pulse measure unit) were used for applying programming signals and sensing/read. A chip of OxRAM devices was wire bonded with NMOS transistor(s) to realize SLIM bitcell. NMOS transistors from CD4007UB CMOS dual complementary pair plus inverter IC were used. During experiments, V_*G*_ was supplied from external power supply and drain voltage (V_*D*_)/source voltage (V_*S*_) was applied using Keithley 4210 high power SMU. While characterizing NMOS transistors, a passive resistor of 1 kΩ was placed in series.

## Supplementary information

Supplementary Information.

## Data Availability

The data that support the plots within this paper and other findings of this study are available from the corresponding author upon reasonable request.
