# Supplementary material for: SLIM: Simultaneous Logic-in-Memory Computing Exploiting Bilayer Analog OxRAM Devices
Source: Sci Rep. 2020 Feb 13;10:2567. doi: 10.1038/s41598-020-59121-0 (PMC7018944; doi:10.1038/s41598-020-59121-0)
Supplement: Supplementary file 1 — Supplementary Information. [file 41598_2020_59121_MOESM1_ESM.pdf]

Supplemental Information

SLIM: Simultaneous Logic-in-Memory Computing Exploiting Bilayer Analog OxRAM Devices

[1]Sandeep Kaur Kingra, [1]Vivek Parmar, [2]Che-Chia Chang, [2]Boris Hudec, [2]Tuo-Hung Hou, [1*]Manan Suri
[1]Department of Electrical Engineering, Indian Institute of Technology-Delhi, Hauz Khas, New Delhi, 110016, India.
[2]Department of Electronics Engineering and Institute of Electronics, National Chiao Tung University, Hsinchu, 300, Taiwan.

S1 Repeatable Analog conductance tuning characteristics for V_{READ} = -0.4 V/-1.5 V.

Consecutive SET/RESET pulses with amplitude (3 V/-5.5 V)/(3 V/-3 V) were applied using Keithley 4225 PMU. After each SET/RESET pulse, a Read signal, V_{READ} = -0.4/-1.5 V was applied. The current through the device was measured and corresponding conductance value was calculated. High reproducibility of the electrical characteristics with small variations can be attributed to the high uniformity of dielectric films deposited by PE-ALD and the non filamentary switching nature of these OxRAM devices. Electrode's line resistance in the cross-bar array layout used is partially responsible for the D2D resistance variations observed.

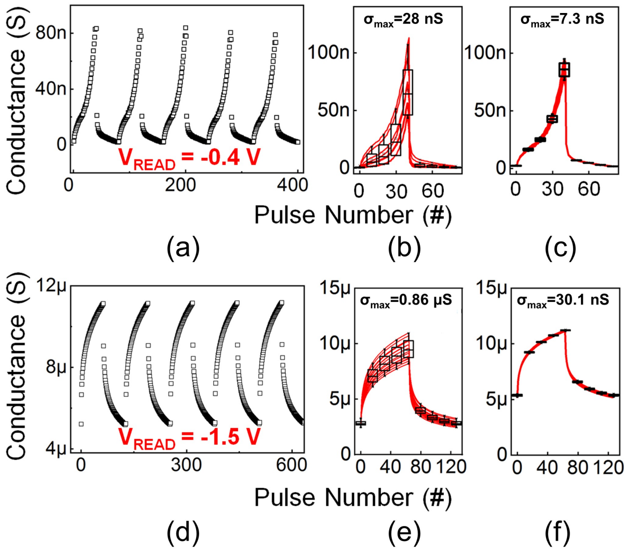

Figure S1. Repeatable analog conductance tuning characteristics observed in the OxRAM device using identical SET and RESET pulses trains for (a) V_{SET} = 3 V (10 ms) , V_{RESET} = -5.5 V (10 ms) and V_{READ} = -0.4 V. Effect of (b) D2D variability (from 10 devices), σ_{max} = 28 nS (at mean= 57.3 nS), (c) C2C variability (for 30 cycles) , σ_{max} = 7.35 nS (at mean= 79.6 nS) has been observed. Similar characteristics for (d) V_{SET} = 3 V (1 ms), V_{RESET} = -3 V (5 ms) and V_{READ} = -1.5 V, along with (e) D2D variability (from 16 devices), σ_{max} = 0.86 μS (at mean= 9.49 μS) and (f) C2C variability (from 30 cycles), σ_{max} = 0.031 μS (at mean= 11.2 μS) has been observed experimentally.

S2 Transistor Characteristics

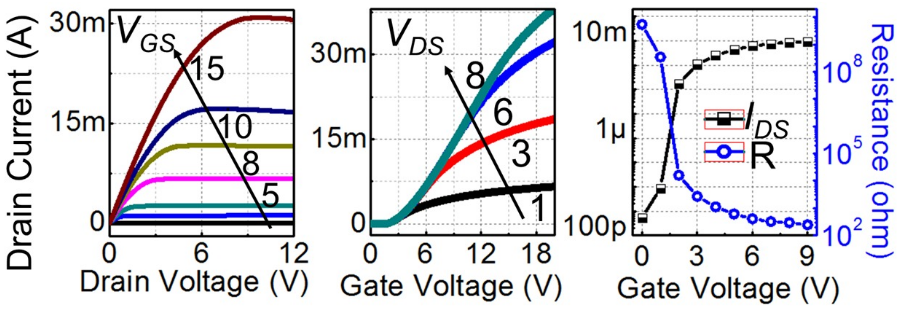

Figure S2. Experimentally measured NMOS characteristics: I_D-V_{DS} plot, I_D-V_{GS} plot and relationship between NMOS transistor enforced compliance current (Y1 axis) and ON resistance (Y2 axis) with gate voltage (V_{DS} = 3 V).

S3 Experimental Setup used in this study

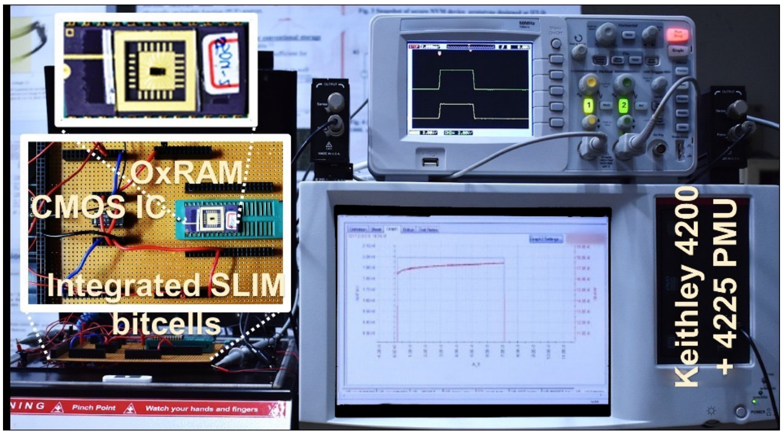

Figure S3. Experimental setup used for SLIM characterization. It shows integrated 2T-1R SLIM bitcell, CMOS chip, OxRAM chip and parameter analyzer used for measurements.

S4 Device parameters used for SLIM application analysis

Table S1. Device parameters used for 1T-1R/2T-1R SLIM bitcell array based application analysis[27,28].

Parameter	Value
Switching Energy	10 pJ
Read Energy	0.25 pJ
Switching Latency	10 ns
MAT configuration	8×8[1], 32×32[2]
MATs per bank	32
Total banks	16

[1] for edge detection, [2] for BNN

S5 BNN performance estimation for SLIM bitcell array

BNN mapping on SLIM array, uses two levels of mapping: Layer based mapping followed by Operation based mapping. This ensures that all computations of one layer are completed before proceeding with the next. Operation scheduling is performed such that XNOR operations are completed prior to POPCOUNT computation by simultaneously scheduling them across all MATs. Since hidden layer uses 8-bit inputs as shown in Figure 11(a), XNOR operations need to be performed for all bit-planes of input separately for each hidden layer neuron. We would also like to point out that SLIM MATs are not shared between neurons, which simplifies scheduling of POPCOUNT operations. For first stage POPCOUNT estimation based on LUT, 16-bit inputs are fed to SRAM based LUT and 4-bit POPCOUNT value is obtained. For power estimation, we used a 28nm 32kb SRAM from literature as reference[34]. Due to this, a row (32-bit) computed on SLIM MAT can be used as an input to two SRAM based LUT modules. The computed first level POPCOUNT output from LUTs, is then mapped back onto new SLIM MATs. In order to get POPCOUNT of each neuron, all 4-bit intermediate outputs are accumulated using a hierarchical adder tree (shown in Figure S4(c)) with increasing bit-width at each level. As a result, we obtain POPCOUNT for 1 bit-plane for one neuron. Once all 8 POPCOUNT values are available, a Shift-Add based summation is performed. The offset is then subtracted to obtain actual sign value corresponding to the activation of neurons. For mapping BNN-MLP operations on CPU, Memory operations are performed by LOAD and STORE to get 64-bit operands. XOR and NOT instructions are used to compute bit-wise XNOR followed by a first level POPCOUNT output implemented using POPCNT instructions. Final POPCOUNT output is derived using ADD operations and offset adjusted using SUB. Final activation value is decided using CMP instruction. For hidden layer output adjustment to combine bit-planes, SHL is used alongside ADD. The reduction in EDP benefit (as shown in Table 6) in comparison to edge detection application is because of the POPCOUNT operation which significantly impacts delay. The POPCOUNT computation is serial in nature as each level of the hierarchical adder tree needs to be computed in sequence. Hence computation delay is dependent on network layer input size which determines the number of levels for summation.

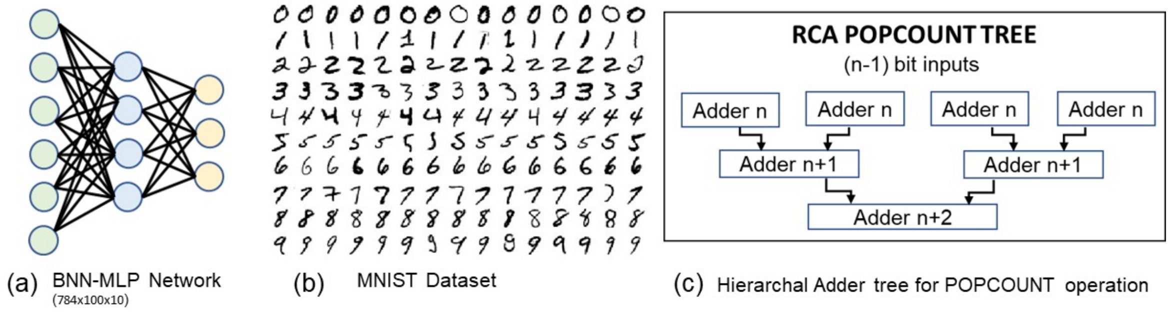

Figure S4. (a) Network topology with 784 input neurons, 100 hidden neurons and 10 output neurons, (b) MNIST dataset used for training the network in our study, and (c) Hierarchical POPCOUNT tree for neuron accumulation realized using RCA of increasing bit width.

S6 Endurance Analysis for BNN-MLP application

In order to investigate the impact of OxRAM device endurance on SLIM based computation, we performed an array size based analysis. In this analysis, the SLIM array size was varied from 1 kb to 8 Mb (where each array comprises of 32×32 SLIM MATs) to solve the BNN-MLP application. As shown in Figure S5, the maximum device endurance requirement drops significantly with increase in the array size. Even for worst case (i.e. single SLIM MAT to run full BNN), the max write ops/device were estimated ≈ 8000, well below typical OxRAM device endurance reported in literature[37]. Further, since we are proposing SLIM mainly for high-density storage solutions (GBs/TBs) the impact of device endurance will be mitigated.

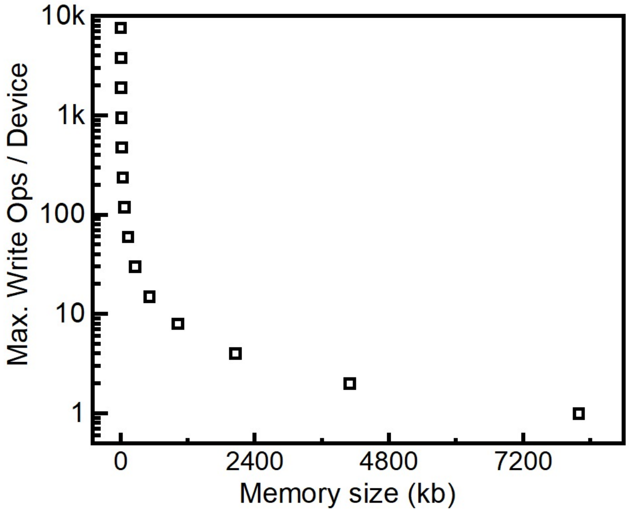

Figure S5. Endurance requirement analysis for performing a single BNN inference operation with varying SLIM array sizes. Significant decrease in OxRAM maximum write-hits per device is observed with increase in the array size.
